# *Synechococcus elongatus* Argonaute reduces natural transformation efficiency and provides immunity against exogenous plasmids

**DOI:** 10.1128/mbio.01843-23

**Published:** 2023-10-04

**Authors:** Arnaud Taton, Tami S. Gilderman, Dustin C. Ernst, Carla A. Omaga, Lucas A. Cohen, Camilo Rey-Bedon, James W. Golden, Susan S. Golden

**Affiliations:** 1 School of Biological Sciences, University of California, San Diego, La Jolla, California, USA; 2 Center for Circadian Biology, University of California, San Diego, La Jolla, California, USA; UCLA School of Medicine, Los Angeles, California, USA

**Keywords:** cyanobacteria, horizontal gene transfer, Argonaute, cyanophage, plasmid, genetic engineering

## Abstract

The cyanobacterium *Synechococcus elongatus* PCC 7942 produces an active prokaryotic Argonaute nuclease, SeAgo, whose function is unknown. Here, we show that SeAgo reduces natural transformation and prevents the maintenance of RSF1010 replicons in *S. elongatus*. In addition, a Cas4-like nuclease and two other proteins, UvrD and RecJ_cy_ (cyanobacterial lineage), were found to reduce the transfer or maintenance of RSF1010 replicons. Like other prokaryotic Argonautes, our results indicate that SeAgo provides defense against invading DNA. An *S. elongatus ago* deletion strain shares the same morphology, growth rate, and circadian gene expression as the wild type, has higher transformation efficiency, and enables the use of RSF1010-based plasmids for genetic engineering.

**IMPORTANCE:**

*S. elongatus* is an important cyanobacterial model organism for the study of its prokaryotic circadian clock, photosynthesis, and other biological processes. It is also widely used for genetic engineering to produce renewable biochemicals. Our findings reveal an SeAgo-based defense mechanism in *S. elongatus* against the horizontal transfer of genetic material. We demonstrate that deletion of the *ago* gene facilitates genetic studies and genetic engineering of *S. elongatus*.

## INTRODUCTION

Cyanobacteria comprise an important group of prokaryotes that arose early during the evolution of eubacteria (1). As the first organisms to perform oxygenic photosynthesis, cyanobacteria were responsible for the oxygenation of the Earth’s atmosphere during the Precambrian. Today, cyanobacteria still hold an essential role as primary producers, generating a significant fraction of the Earth’s oxygen and contributing to major biogeochemical cycles. Over at least 2.7 billion years of evolution, cyanobacteria have developed diverse morphologies and metabolic capabilities. The evolution of cyanobacteria includes the appearance of diazotrophy, multicellularity, and cell specialization (2, 3).

As is common among prokaryotes, the evolution of cyanobacteria has been strongly influenced by horizontal gene transfer (HGT) mediated by conjugation, natural competence, and phage transduction (4). The acquisition of novel genes through HGT can provide fitness benefits, but exposure to non-native DNA also requires strategies to combat fitness-compromising invasive mobile genetic elements (MGEs), particularly phages (5). In addition to the physical barriers provided by the cell wall, S-layer, and exopolysaccharide envelope, cyanobacteria employ innate and adaptive defense strategies, also referred to as immunity, common among prokaryotes to defend against MGEs (6).

Bacterial innate immunity largely relies upon restriction-modification (RM) systems. These are found in most cyanobacteria, including strains harboring some of the highest numbers of RM systems known (7, 8). For example, single strains of *Arthrospira* and *Microcystis* have up to 70 and 86 RM enzymes, respectively [REBASE (7)]. Other innate immunity systems include DNA phosphorothioation and bacteriophage exclusion that are reminiscent of the RM systems but have not been reported in cyanobacteria (5). Finally, prokaryotic Argonaute (pAgo) proteins are also categorized as innate immunity components. pAgo proteins protect various bacteria against invasive MGEs, and pAgo homologs are encoded in cyanobacterial genomes (9, 10). Adaptive immunity encompasses CRIPSR-Cas systems, which are abundant and diverse among cyanobacteria (11, 12).

pAgo proteins have been categorized into three groups: short, long-A, and long-B pAgo proteins based on their domain composition and conservation. Only long-A pAgos include all four characteristic domains (N-, PAZ, MID, and PIWI) of the Ago family and are predicted to be catalytically active (13). Sharing a mechanism similar to the CRISPR-Cas system, pAgos generate short RNA or DNA oligonucleotides from invasive MGEs. Although the oligonucleotides are not stored in genomic arrays as for the CRISPR-Cas system, they are used as guides by pAgos to recognize and destroy new invaders or other MGE copies (14). The roles and mechanisms of action of pAgo have been largely elucidated in *Clostridium butyricum* (CbAgo) (15). CbAgo targets multicopy genetic elements (rRNA operons, IS elements), induces DNA interference between homologous sequences, and triggers DNA degradation at double-strand breaks. Notably, CbAgo suppresses the propagation of invader DNA such as plasmids and phages, acting as an MGE defense system.

The cyanobacterial genetic model strain *Synechococcus elongatus* PCC 7942 encodes a long-A pAgo (SeAgo) that includes all characteristic domains of the Ago family. The closest active and well-studied pAgo homolog of SeAgo is TtAgo from *Thermus thermophilus*. SeAgo is structurally similar to and shares 29% sequence identity (46% similarity, including conservative substitutions) with TtAgo, which reduces natural transformation and enables the completion of chromosome replication in *T. thermophilus* (13, 16, 17). SeAgo was identified as the highest negative determinant of natural transformation in a genetic screen (18), and its activity was recently characterized (19). SeAgo is a DNA-guided nuclease that preferentially targets single-stranded DNA (ssDNA), with non-specific guide-independent activity on double-stranded DNA. SeAgo is associated with small DNA guides whose formation depends on its own endonuclease activity and co-purifies with single-strand DNA-binding protein. The SeAgo-loaded DNA guides are derived from diverse genomic locations, with an enrichment for the predicted sites of chromosomal replication initiation and termination (19).

Here, we investigated the role of SeAgo for natural transformation as well as maintenance of RSF1010-based plasmids in *S. elongatus*. In addition, we examined the potential of a markerless deletion of the *ago* gene as a useful genetic background for further genetic studies in *S. elongatus*.

## RESULTS AND DISCUSSION

### Distribution of Argonaute in the cyanobacterial phylum

Homologs of pAgo are encoded in genomes of cyanobacteria other than *S. elongatus* (9). To obtain a comprehensive picture of the distribution of pAgo across the cyanobacterial phylum, the 23 long-A pAgo sequences previously identified in cyanobacterial genomes by Swarts et al. (9) were used in BLAST searches to query the NCBI non-redundant protein sequence database (nr). One hundred sixty cyanobacterial pAgo homologs were identified from diverse genera including members of each systematic subsection (20). However, only a fraction (20/160) of those long-A pAgo homologs carry the conserved PAZ domain and the four conserved residues of the catalytic tetrad in the PIWI domain (DEDX, where X is D, H, or K) that are expected for a catalytically active pAgo. These were found largely in unicellular cyanobacteria of subsection I, including other *S. elongatus* isolates, relatively close lineages of *Synechococcus* and *Thermosynechococcus*, genetically distant *Synechococcus* species such as strain PCC 7002, as well as *Synechocystis* and *Gloeothece* species. Predicted active pAgos with a conserved PAZ domain and the DEDX tetrad were also identified in unicellular cyanobacteria of subsection II such as *Stanieria* and *Pleurocapsa* genera. In contrast, among genomes of filamentous cyanobacteria, predicted active pAgos were identified in only two sequences, which were assembled from metagenomic data; these were not included in further analyses. Similar to other prokaryotic immunity systems, such as restriction and CRISPR-Cas systems, pAgos were probably acquired by horizontal gene transfer (9). To investigate the origin of pAgo within the cyanobacterial phylum, the evolutionary history of cyanobacterial pAgos was compared with that of the 16S rRNA gene.

**Fig 1:**
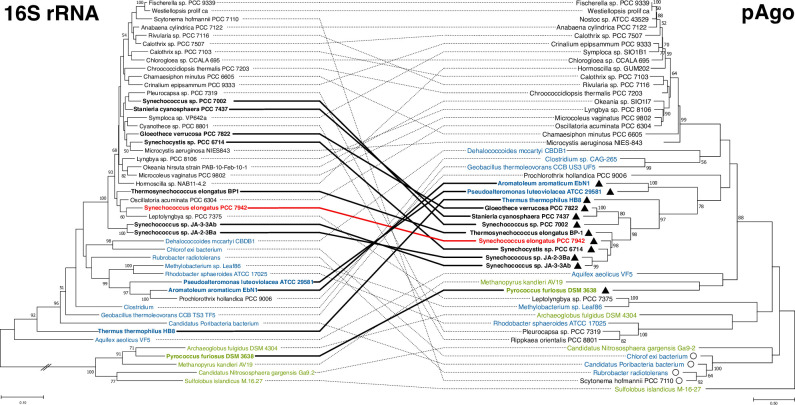
Evolutionary history of long-A pAgo in cyanobacteria. 16S rRNA gene and pAgo phylogenetic trees inferred by maximum likelihood. Trees with the highest log likelihood are shown (16S rRNA: −19103.12, pAgo: −10635.27). A discrete gamma distribution was used to model evolutionary rate differences among sites (five categories, 16S rRNA: 0.5225, pAgo: 3.5185). This analysis included 1,365 positions for the 16S rRNA gene and 179 positions for pAgo. Trees are drawn to scale, with branch lengths measured in the number of substitutions per site. The percentage of trees (*n* = 500) in which the associated taxa clustered together is shown next to the branches. Black triangles and solid black and red connecting lines mark predicted catalytically active long-A pAgo proteins. Open circles mark pAgo proteins without a PAZ domain but with HATPase and predicted C-terminal DNA-binding domains. Cyanobacteria are colored in black, except *S. elongatus* colored in red; other bacteria are colored in blue, and archaea, in green.

The phylogenetic analyses (Fig. 1) show that all predicted active pAgos are grouped in a monophyletic cluster branching from the same node as *T. thermophilus*, suggesting a common ancestor, whereas other pAgos have originated from alternative lineages. The analyses reveal that active pAgos are not well conserved in the cyanobacterial phylum. Phylogenetic analysis of the 16S rRNA gene shows that the cyanobacteria harboring a long-A pAgo belong to different genera and phyla. The phylogenetic clustering, coupled with the small number of active pAgos found among cyanobacteria, supports the hypothesis of horizontal inheritance.

### SeAgo reduces natural transformation

Our study of SeAgo was motivated by a genetic screen of a dense library of *S. elongatus* randomly barcoded transposon mutants (hereafter *S. elongatus* RB-TnSeq library) (21), which revealed that the loss of function of SeAgo had the highest positive effect on natural transformation of *S. elongatus* (18). This result suggested that knock-out (KO) strains of the *ago* gene would result in higher transformation efficiencies of *S. elongatus*. To test this hypothesis, transformation assays were performed in two independent insertional KO strains of the *ago* gene (*ago*::Tn*5*-BB11 and *ago*::Tn*5*-HH1) and a control kanamycin-resistant strain of *S. elongatus* that carries a Tn*5* insertion at neutral site 2 (NS2) (NS2::Tn*5*-K12). Cultures of each strain were transformed with suicide plasmids that integrate into *S. elongatus* chromosomes and provide selection with either the *aadA* gene at neutral site 1 (NS1) conferring spectinomycin and streptomycin resistance (NS1::Sp^R^+Sm^R^) or the *aacC1* gene at neutral site 3 (NS3) conferring gentamycin resistance (NS3::Gm^R^). Transformation assays were performed on strains grown under 12 h:12 h light:dark (LD) cycles at 2 h following the return of light (dawn) and at 2 h after the onset of darkness (dusk) when the efficiency of natural transformation is either minimal or maximal, respectively (18).

**Fig 2:**
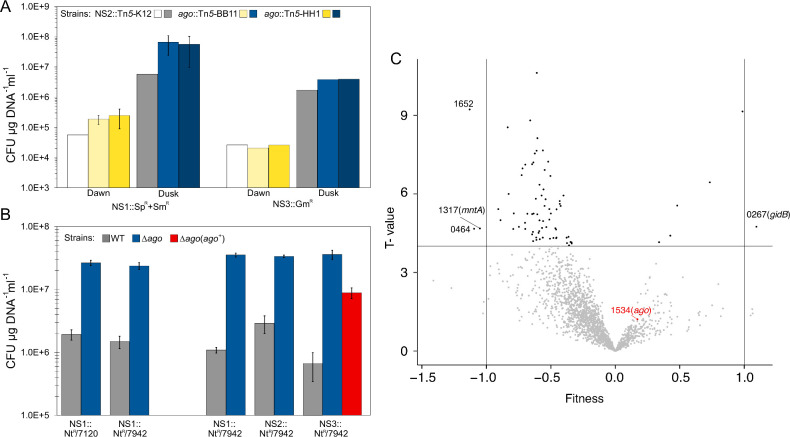
SeAgo reduces natural transformation efficiency but does not affect conjugation. (**A**) Natural transformation efficiency, expressed as the number of antibiotic-resistant CFUs, at dawn and at dusk for two independent insertion KO strains of *ago* (*ago*::Tn*5*-BB11 and *ago*::Tn*5-*HH1) and a control strain (NS2::Tn*5-*K12). Transformations were with neutral-site plasmids pAM5329 and pAM5328 that recombine at NS1 (NS1::Sp^R^+Sm^R^, *n* = 2 for the *ago* KOs) and NS3 (NS3::Gm^R^) in the chromosome. Data are plotted on a log10 scale as mean values ± standard deviation (SD). (**B**) Natural transformation efficiency for WT and an *ago* markerless deletion (AMC2664). Transformations were performed with neutral-site plasmids pAM4920 and pAM4921 that carry different Nt^R^ genes and recombine at NS1 (NS1::Nt^R^/7120 and NS1::Nt^R^/7942) and plasmids pAM5602, pAM5605, and pAM5607 that carry the same Nt^R^/7942 gene but recombine at three different neutral sites (NS1::Nt^R^/7942, NS2::Nt^R^/7942, and NS3::Nt^R^/7942). Additionally, a complemented strain, ∆*ago*(*ago^+^*), was transformed with plasmid pAM5607. Data are plotted on a log10 scale as mean values ± SD, *n* = 3 for the *ago* KOs and *n* = 2 for the WT. (**C**) RB-TnSeq library analysis of gene transfer by conjugation. Fitness values and confidence levels (T-values) of barcoded transposon mutants of the RB-TnSeq library following the conjugal transfer of neutral-site plasmids pAM5329 (NS1::Sp^R^+Sm^R^) and pAM5544 (NS2::Nt^R^/7942). Fitness values were calculated for 1,893 genes from three independent experiments, representing a total of 143,926 distinct mutants obtained under selective conditions and 139,579 mutants under control conditions without selection. Selected data points were labeled with *S. elongatus* locus tag (Synpcc7942_) numbers and gene names in parentheses.

At dawn, the circadian minimum for transformation in the wild type (WT), the transformation efficiencies of the *ago* KO strains were up to four times higher than for the control strain with the NS1::Sp^R^+Sm^R^ integration plasmid. However, transformation efficiencies were similar between KO and control strains for the plasmid that integrates a Gm^R^ gene at NS3 (Fig. 2A). At dusk, the permissive time for transformation, the transformation efficiencies of the *ago* KO strains were up to 10 times higher than for the control strain with the NS1::Sp^R^+Sm^R^ plasmid and twice as high with the NS3::Gm^R^ plasmid (Fig. 2A). These results confirmed that SeAgo inhibits the transformation of *S. elongatus* at dawn and dusk and explains the fitness advantage of *ago* KO strains in RB-TnSeq experiments with selection for transformation efficiency (18).

The differences in transformation efficiencies between assays targeting distinct neutral sites or using different antibiotic resistance genes (Fig. 2A) could be due to the location in the chromosome targeted for homologous recombination or the nucleotide composition of the plasmid DNA. Therefore, we targeted different neutral sites with the same insert and different antibiotic resistance genes at the same neutral site. We used a single nourseothricin-resistance gene that was codon optimized for *S. elongatus* for transformation assays at three neutral sites (NS1::Nt^R^/7942, NS2::Nt^R^/7942, and NS3::Nt^R^/7942). We also targeted NS1 using a different Nt^R^ gene with a distinct sequence that was optimized for *Anabaena* PCC 7120 (NS1::Nt^R^/7120). The coding sequences of the two Nt^R^ genes were 20% dissimilar with GC contents of 61% and 41% for *S. elongatus* and *Anabaena* optimized sequences, respectively (Fig. S1). To avoid potential polar effects associated with the Tn*5* insertions in the *ago*::Tn*5*-BB11 and *ago*::Tn*5*-HH1 mutants, we constructed a markerless deletion strain of the *ago* gene (∆*ago*). We performed transformation assays at NS3 (NS3::Nt^R^/7942) in the ∆*ago* mutant strain and a complemented strain, designated ∆*ago*(*ago^+^*).

For these new assays, transformation efficiencies were 1 to 2 orders of magnitude higher in the ∆*ago* strain compared to the WT strain (Fig. 2B). For the ∆*ago* strain, similar levels of transformation were observed for the three neutral sites and for both versions of the Nt^R^ gene targeted at NS1. The complementation of *ago* (∆*ago*(*ago^+^*)) reduced the transformation efficiency by more than 75% but did not fully restore the WT phenotype. Thus, SeAgo strongly inhibits transformation by DNA that recombines into the *S. elongatus* chromosome. There were small differences in transformation efficiencies of the WT at the three different neutral sites (Fig. 2B; Fig. S2). These results suggest that SeAgo may differentially target DNA elements that contain different regions of homology with the chromosome. However, the similar levels of transformation observed for the two different versions of the Nt^R^ gene in the WT and ∆*ago* strains show that SeAgo does not differentially target these two sequences.

In the WT strain, the lowest transformation efficiency was observed at NS3, which lies within a cryptic prophage (chromosome: 711256..759990) (22). NS3 is in a region of the *S. elongatus* chromosome that is more heavily sampled for DNA guides by SeAgo (19). We conclude that the prophage-containing DNA is more likely to be cleaved by SeAgo guide-dependent activity, consistent with a defense function for SeAgo.

### SeAgo does not affect DNA transfer by conjugation

In our previous work that identified *ago*, we screened the *S. elongatus* RB-TnSeq library for genes that affect natural transformation (18). The library comprises a pool of ~250,000 individual barcoded transposon mutants distributed across the *S. elongatus* 2.75-Mb genome. Under standard laboratory condition, 764 of the 2,723 genes were deemed essential for phototrophic growth because mutants of these genes are absent or largely depleted from the library (21). Under selective pressure, particular mutants become depleted from the pool or overrepresented depending on whether the mutation is detrimental or beneficial for fitness. Therefore, comparison of barcodes before and after the library has been grown under selective and control conditions provides an efficient method to identify genes that contribute to fitness in a particular condition (23). Here, we screened the RB-TnSeq library using conjugal transfer of a selectable marker to identify potential *S. elongatus* defense mechanisms against DNA uptake by conjugation. Plasmid DNA carrying different selectable markers that recombine into neutral sites 1 and 2 (NS1::Sp^R^+Sm^R^ and NS2::Nt^R^/7942) were conjugated into the *S. elongatus* RB-TnSeq library. Mutant colonies were collected as pools from selective and non-selective control conditions, and the barcodes were sequenced and quantified. Three experiments were performed, and the relative abundance of mutants for each gene in both conditions was reported in a single analysis as fitness with a corresponding level of confidence (T-value) (Fig. 2C; Data Set S1). The fitness value, based on reproduction of cells that carry an insertion in a particular locus, describes how the loss of function of a particular gene affects the conjugal transfer of DNA and its recombination into the chromosomes of *S. elongatus*. The T-value describes the significance of the fitness difference between experimental and control conditions (18, 24, 25).

The RB-TnSeq results did not identify any genes with strong positive or negative fitness for conjugation and show that the loss of function of SeAgo did not produce a fitness advantage for conjugal transfer of DNA into *S. elongatus*. These results indicate that the conjugal transfer of DNA into *S. elongatus* does not require any non-essential genes in the recipient cells. These results also suggest that *S. elongatus* does not have a defense against the conjugal transfer of DNA. During conjugation, the transferred ssDNA strand is re-circularized and rapidly converted to dsDNA (26), whereas in natural transformation, ssDNA enters the cell and remains mostly single stranded until it integrates into the chromosome (27). Because SeAgo preferentially targets ssDNA (19), this difference may explain why SeAgo inhibits natural transformation but not conjugation.

The disruption of four genes resulted in low but significant fitness changes with fitness values below −1 or above +1 and an absolute T-value above 4 (false discovery rate <0.001) (Fig. 2C; Data Set S1). The loss of function of three of these genes decreased conjugation efficiencies but still yielded many exconjugants. These genes encode a YbaB/EbfC family nucleoid-associated protein (Synpcc7942_0464), an rSAM superfamily protein (Synpcc7942_1652), and an ATPase, MntA (Mn^2+^ transport). The loss of function of the *gidB* gene, which encodes a homolog of RsmG (ribosomal RNA small subunit methyltransferase G) increased exconjugant yield. An enrichment analysis into functional categories of the additional genes whose loss of function affected conjugal transfer in *S. elongatus* with small but significant fitness changes (fitness values between −1 and 0 with an absolute T-value >4) did not reveal any specific cellular pathway required for conjugal transfer into *S. elongatus* (Data Set S1).

### SeAgo interferes with the maintenance of broad-host-range RSF1010 replicons

Genetic engineering of *S. elongatus* for complementation of gene knockouts, gene over-expression, and the expression of heterologous genes or reporter fusions is typically carried out by transformation with DNA designed to recombine into chromosomal neutral sites (28). Alternatively, shuttle plasmids based on the *S. elongatus* small plasmid pANS and engineered to also replicate in *Escherichia coli* can be useful as vectors for ectopic or heterologous gene expression (29 – 31). Broad-host-range plasmids based on RSF1010 are known to be stably maintained in many Gram-negative bacteria, including various strains of cyanobacteria, and some Gram-positive bacteria (32 – 34). However, we and others have found that RSF1010-based plasmids are not maintained well in *S. elongatus*. The conjugal transfer of RSF1010-based pSL2680 derivatives (35) used for CRISPR-based genome engineering showed some success in *S. elongatus* (36), but only tiny colonies were obtained after conjugation, and several passages of these transconjugant colonies on selective plates were required to obtain strains that grew well and carried the expected CRISPR edits. These observations together with the role of pAgo in suppression of plasmid propagation in *C. butyricum* (15) led us to hypothesize that SeAgo may interfere with the maintenance of RSF1010-based plasmids in *S. elongatus*.

**Fig 3:**
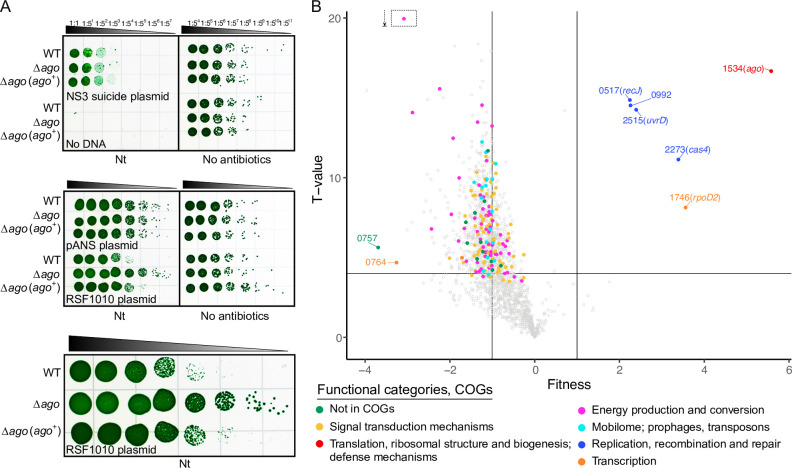
Maintenance of RSF1010 replicons in *S. elongatus*. (**A**) Growth of transconjugants with or without nourseothricin after conjugal transfer of a neutral-site plasmid pAM5554 (NS3::Nt^R^) and two replicating plasmids, pAM4971 (pANS-Nt^R^) and pAM4970 (RSF1010-Nt^R^), into WT, ∆*ago* (AMC2774), and ∆*ago*(*ago^+^*) (AMC2775) strains. The bottom panel is an enlargement of the plating assay for pAM4970. The spot-plating assays were performed as 1:5 dilution series in triplicate, and a representative assay is shown. (**B**) RB-TnSeq library analysis with an RSF1010-based plasmid. Fitness values and confidence levels of barcoded transposon mutants following the conjugal transfer of plasmid pAM5407 (RSF1010-Sp^R^+Sm^R^) relative to that of the control plasmid pAMT025 (pANS-Sp^R^+Sm^R^), which replicates well in *S. elongatus*. Fitness values were calculated for 1,886 genes, from two independent experiments, representing a total of 86,431 distinct mutants obtained after the conjugal transfer of pAM5407 and 120,214 mutants after the conjugal transfer of pAMT025. Selected data points were labeled with *S. elongatus* locus tag (Synpcc7942_) numbers and gene names in parentheses.

To test this hypothesis, three plasmids, a suicide plasmid that recombines at NS3, a plasmid that carries the pANS replicon, and an RSF1010-based plasmid, were conjugated into WT, ∆*ago*, and ∆*ago*(*ago^+^*) strains cured of the *S. elongatus* native pANS plasmid (30). Conjugation of a plasmid that recombines at NS3 or a pANS-based plasmid produced similar numbers of colonies for the three strains, confirming that SeAgo does not interfere with the transfer of DNA by conjugation (Fig. 3A). As expected, conjugation of the RSF1010-based plasmid into the WT and complemented ∆*ago*(*ago^+^*) strains produced only tiny, generally not sustainable, colonies that cannot grow when transferred to a fresh selective plate. However, conjugation of the RSF1010-based plasmid into the ∆*ago* strain produced similar numbers of colonies as did the pANS-based plasmid, which indicates that the RSF1010-based plasmid is maintained well in the absence of SeAgo. Conjugation of the pANS- and RSF1010-based plasmids into the ∆*ago* strain produced more than a thousand times more colonies than did the NS3 suicide plasmid. These results indicate that the number of transconjugant colonies was limited by the rate of homologous recombination into the chromosome. Altogether, these results show that SeAgo interferes with the maintenance of RSF1010-based plasmids in *S. elongatus* and further support the role of SeAgo in the defense against foreign DNA.

To determine whether genes other than *ago* affect the conjugation or maintenance of RSF1010-based plasmids in *S. elongatus*, we introduced an RSF1010-based plasmid carrying an Sp^R^+Sm^R^ gene into the *S. elongatus* RB-TnSeq library by conjugation (Fig. 3B; Data Set S2). In a control conjugation, we introduced a pANS-based plasmid that carries an Sp^R^+Sm^R^ gene. The loss of function of six genes, identified with high confidence (fitness values > 1 and absolute T-values > 4) positively affected conjugation or maintenance of the RSF1010-based plasmid. These genes include *ago*, as expected, and *cas4*, *uvrD*, *recJ_cy_* (a *recJ* paralog found in the cyanobacterial lineage), *rpoD2*, and the Synpcc7942_0992 gene encoding a conserved hypothetical protein with a PD-(D/E)XK endonuclease-like domain. Genes that encode long-A pAgos with intact catalytic sites occasionally cluster with genes encoding a Cas4-like protein (9). Cas4 is a CRISPR-associated nuclease/helicase that is often found alone in small-genome bacteria and archaea that typically lack full CRISPR-Cas systems, suggesting that Cas4 may perform uncharacterized defense or repair functions (37). UvrD is an ATP-dependent helicase, and RecJ_cy_ is homologous to the RecJ family of single-stranded DNA-specific exonucleases. UvrD and RecJ are involved in the resolution of stalled replication forks in *E. coli* and *T. thermophilus* (17, 38). Both proteins have paralogs in *S. elongatus,* PcrA (Synpcc7942_2168) and RecJ (Synpcc7942_1886), respectively, which may have redundant or overlapping functions. TtAgo and CbAgo are involved in chromosome and plasmid replication (15, 17); the identification of UvrD and RecJ_cy_ in our *S. elongatus* RB-TnSeq screen suggests that SeAgo may be involved in these processes as well. RpoD2 (also known as SigA2 and SigB) is a group 2 sigma factor whose loss alters the expression of 51% of circadian-regulated mRNAs (39). The loss of function of each of these six genes also benefits natural transformation (18). Understanding the role of these genes in these processes will require additional studies.

Analysis of RB-TnSeq data for the conjugation of an RSF1010-based plasmid compared to a pANS-based plasmid showed negative fitness for almost the entire population of mutants (Fig. 3B; Data Set S2). This result indicates an overall difference in the growth of the experimental and control populations under antibiotic selection for the plasmid, consistent with failure of RSF1010-based plasmids to be maintained well in *S. elongatus*. Mutants defective for hundreds (708) of genes were notably impaired (fitness values <−1 and absolute T-values >4), presumably because these genes’ loss affects growth under stress. Consistent with a stress response, this set of genes was enriched for the mobilome (phages and transposons) (Fig. 3B; Data Set S2). RB-TnSeq mutations in all genes of the *S. elongatus* 49-kb prophage (Synpcc7942_0728–Synpcc7942_0765) (22) had decreased fitness in this experiment, suggesting that the prophage may have been triggered to excise from the chromosome, resulting in cell death (and hence, lack of replication, which underlies the fitness calculation). The loss of function of the prophage genes Synpcc7942_0757, which encodes a hypothetical protein, and Synpcc7942_0764, which encodes the phage immunity repressor protein C gene, resulted in strongly impaired fitness (Fig. 3B; Data Set S2). These results were not investigated further in the current study.

### Selection for maintenance of RSF1010-based plasmids yields second-site mutations in the *ago* gene

**Fig 4:**
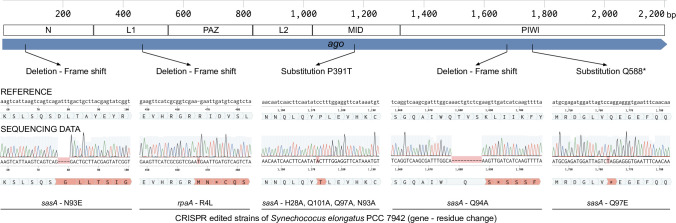
Second-site mutations in *ago* after selection for transconjugant strains that carry an RSF1010-based plasmid. Eight strains were engineered independently to carry a single substitution in SasA or RpaA using CRISPR-Cas12a carried on an RSF1010-based plasmid. The figure depicts a genetic map of *ago*, the locations and types of the second-site mutations in *ago*, the reference sequence for WT *ago* and the sequencing data for the engineered strains around the mutation sites, the amino acid sequence of SeAgo in the engineered strains, and finally the amino acid substitutions engineered in SasA and RpaA.

As described above, we have used CRISPR-Cas12a carried on an RSF1010-based plasmid for gene-editing experiments to determine the influence of single substitutions in SasA and RpaA on the *S. elongatus* circadian clock (36). Eight independent strains were made, but colonies that carry this plasmid grew slowly for many generations before growing more robustly. We hypothesized that this improved growth after passaging is a result of second-site mutations in *ago* that enable maintenance of the RSF1010-based plasmids. Therefore, the *ago* locus from the eight CRISPR-edited strains that had been through selection for maintenance of RSF1010-based plasmids was sequenced, and each carried a mutation in *ago*. These included three deletions of 1, 4, and 10 nucleotides resulting in frame shifts in the SeAgo sequence, nucleotide substitutions resulting in an amino acid substitution, P391T, in the MID domain, and a stop codon in the PIWI domain (Fig. 4; Fig. S3). Proline residues play important roles in the kinetics of protein folding and their secondary structure (40), and four independent strains carried the same proline substitution. Whole genome resequencing of other isolates from our laboratory that have not been through RSF1010-based selection has not found any mutations in *ago*. These results show that there is strong selection for *ago* mutations in strains of *S. elongatus* selected to carry RSF1010-based plasmids.

### *ago* mutant strains have wild-type morphology, growth, and circadian properties

Our results show that knockout strains of *ago* have advantages over the WT for genetic studies and genetic engineering. These advantages include improved natural transformation and the ability to use RSF1010-based plasmids for complementation, over-expression, heterologous expression, and CRISPR-Cas12a editing. Therefore, we further characterized the phenotype of *ago* mutants to assess their suitability for future experimental work.

We sequenced the genomes of two independent Δ*ago* clonal isolates (AMC2751 and AMC2752). Both ∆*ago* clones had the deletion of the *ago* locus as expected (Tables S1 and S2). The ∆*ago* mutant clones both carried two single nucleotide differences in the *rpaA* and *aas* genes compared to the PCC 7942 GenBank reference sequence; however, we previously have shown that these positions in the published genome sequence came from a mutant clone (22). Two other differences from the GenBank sequence were detected that do reflect single nucleotide polymorphisms (SNPs) based on several published *S. elongatus* genomes and other isolates resequenced in our laboratory, although both were present in the parental AMC2665 culture, cryopreserved since 1988, from which the *ago* gene was deleted in this work. We have recently found, by resequencing, that the 1988 *S*. *elongatus* stock (AMC2665) carries low frequency variant genomes (Table S3), which explains both the mutant GenBank-sequence clone (19) and the SNPs in the *ago*-deletion background. Those SNPs include an additional 1-nt substitution in the *aas* gene and a 1-bp deletion in the intergenic region between the *sbtA* gene (Synpcc7942_1475) and Synpcc7942_1476.

**Fig 5:**
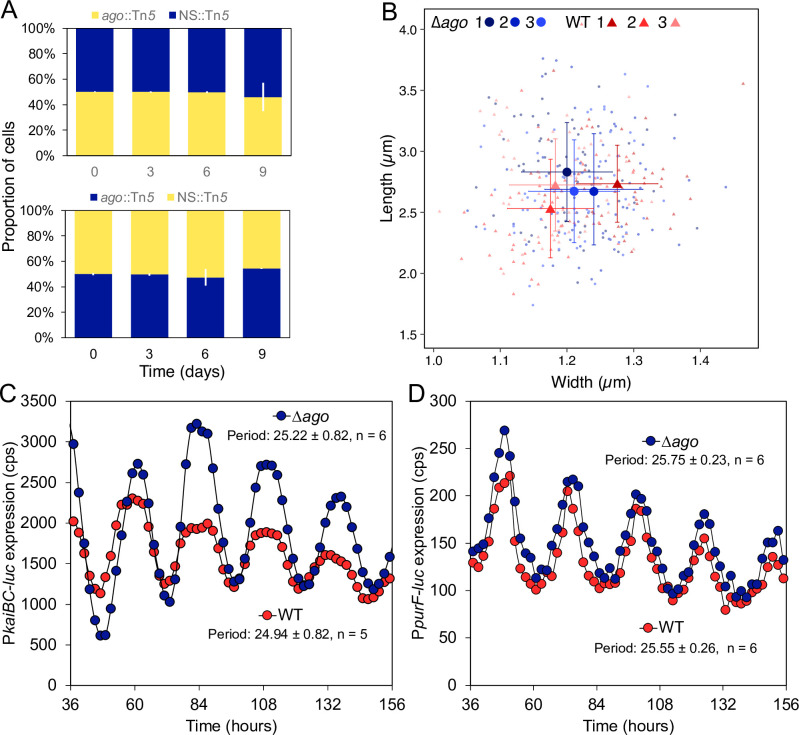
Phenotype of *ago* mutant strains. (**A**) Growth competition assays between *ago*::Tn*5* and control NS3::Tn*5* strains expressing yellow (mVenus) or blue (mTagBFP) fluorescent reporters. The assays were performed in triplicate, and the data were plotted as mean values ± SD. (**B**) Cell sizes of ∆*ago* (AMC2752) and WT strains. Cultures were grown in triplicate, and cell sizes, plotted as mean values ± SD. (**C and D**) Circadian monitoring of bioluminescence from a class 1 (P*_kaiBC_*) and a class 2 (P*_purF_*) promoter. Bioluminescence values were obtained from six replicates, and the data were plotted as mean values. Overall expression levels are more susceptible to environmental variations than are other parameters in these assays; we did not determine whether amplitude varies significantly between these genotypes.

It has been reported that loss of function of *ago* in *S. elongatus* has no effect on growth rate under constant light (19). However, because *S. elongatus* is a model organism for the study of circadian rhythms, we wanted to determine whether the loss of *ago* function affects the growth of *S. elongatus* under diel LD conditions. Growth competition experiments provide a sensitive measure of different relative growth rates (41). We conducted growth competition assays with *ago* KO (*ago*::Tn*5*) and control (NS3::Tn*5*) strains that were engineered to express yellow (mVenus) or blue (mTagBFP) fluorescent reporters. To control for potential effects of the fluorescent reporters on growth, the *ago* KO mutant and control strains were engineered to carry each reporter. Equal numbers of cells of the *ago* KO and control strains expressing different reporters were mixed and grown together in six-well plates for 10 days at 30°C under LD cycles (150 µmol photon m^−2^ s^−1^). The ratio of *ago* KO and control cells was determined by flow cytometry on days 0, 3, 6, and 9. As shown in Fig. 5A, the proportion of cells from each strain changed little during the 10-day time course, indicating that the *ago* mutant and control strains grew at the same rates.

In *T. thermophilus*, TtAgo participates in DNA replication by unlinking concatenate chromosomes (17). Strains with mutant TtAgo grown in the presence of ciprofloxacin, an inhibitor of DNA gyrase subunit A, fail to separate catenated chromosomes, resulting in elongated cells (17). Similarly, the expression of SeAgo in *E. coli* was shown to rescue cell growth and morphology in the presence of ciprofloxacin (42). However, *S. elongatus* WT and *ago* KO strains were shown to have the same growth rates and morphologies in the absence or the presence of ciprofloxacin at sub-minimum inhibitory concentrations (42). We also could not detect a specific phenotype when comparing WT and Δ*ago S. elongatus* strains in 1 µM ciprofloxacin because, under these conditions, cells of both strains were elongated.

We determined the cell sizes of WT and Δ*ago* strains grown under LD cycles as an indication of failure to separate catenated chromosomes. The strains had very similar cells sizes of ~1.2 µm in width by 2.7 µm in length (Fig. 5B). Therefore, under these conditions, the loss of function of SeAgo did not impede cell division or provide evidence that Δ*ago* fails to separate catenated chromosomes. However, these results should be interpreted with caution because cyanobacteria are polyploid (43, 44) and may have different mechanisms for separating catenated chromosomes than the previously tested organisms. The composition and regulation of the cyanobacterial divisome differ from those in other bacteria and, importantly, the chromosome exclusion mechanism required for the formation of the FtsZ-based divisome in *Bacillus subtilis* and *E. coli* is not conserved in *S. elongatus* (45).

To examine whether a deletion of the *ago* gene affects *S. elongatus* circadian rhythms, we constructed bioluminescent reporter strains in the ∆*ago* and WT backgrounds. A firefly luciferase gene driven by either the class 1 P*_kaiBC_* or the class 2 P*_purF_* promoter was integrated into the chromosome at a neutral site for each background. The resulting strains (AMC2747–AMC2750) were grown under LD cycles for 3 days to synchronize the cells, after which the bioluminescence from the luciferase reporter was monitored under constant light for 4 days (Fig. 5C and 5D). The Δ*ago* strains with both reporter systems exhibited rhythmic patterns of bioluminescence that were similar to those of the WT. We did not observe any significant difference in circadian phase or period between the Δ*ago* and WT strains.

### Conclusions

The horizontal transfer of genetic material by natural competence, conjugation, and phage transduction is an important driver in the evolution of prokaryotes. Bacteria use a range of mechanisms to limit the entry of new genetic material, which may provide fitness advantages by introducing novel genes but also poses risks by admitting phages and mobile genetic elements. *S. elongatus* takes up DNA from its environment through natural transformation, but until recently, it was thought to have limited defense strategies against invading DNA, as the genome encodes only three annotated putative RM systems and no CRISPR systems other than one Cas4-like nuclease. We recently showed that natural competence is tightly regulated by the circadian clock in coincidence with darkness, limiting the uptake of DNA from the environment. That study also found that loss of SeAgo increases natural transformation efficiency (18).

In this work, we confirmed that the inactivation of the *ago* gene in *S. elongatus* dramatically increases natural transformation levels. Conjugal DNA transfer is not affected by SeAgo, as determined by recombination of homologous sequences into chromosome neutral sites or a shuttle vector based on a native plasmid replicon. However, SeAgo prevents the maintenance of RSF1010 replicons, and selection for maintenance of RSF1010-based plasmids yields clones with mutations in the *ago* gene. Screening of the *S. elongatus* RB-TnSeq library for mutants that support RSF1010 replicons confirmed that SeAgo interferes with their maintenance and revealed that a Cas4-like nuclease, UvrD, and RecJ_cy_ also interfere with the transfer or maintenance of RSF1010 replicons. The roles of these latter two proteins in DNA replication and repair suggest that SeAgo, like CbAgo and TtAgo, may be involved in chromosome and plasmid replication and acquires its guide DNAs from stalled replication forks (15, 17). If exogenous plasmids such as RSF1010 and some phages do not replicate as well as does the native chromosome, they would be more likely to have stalled replication forks that produce guides for SeAgo nuclease function; this bias in replication efficiency could be exploited by SeAgo as a defense strategy that distinguishes self from non-self. Many species of cyanobacteria encode long-A pAgo proteins, but only a small fraction are predicted to be catalytically active. Previous studies have shown that genes encoding inactive pAgo proteins are often found in the genomic context of various nuclease genes (9, 10). These observations raise further questions about the roles and mechanisms of action of pAgos in the cyanobacterial phylum.

*S. elongatus* (including strains PCC 6301, PCC 7942, UTEX3055, and UTEX2973) is an important model organism for the study of circadian rhythms and other biological processes and for the production of renewable biochemicals and other bioproducts. The elimination of SeAgo activity greatly enhances the efficiency of genetic manipulation of *S. elongatus* and the loss of the *ago* gene does not impair morphology, growth, or circadian behaviors. Thus, the ability to use RSF1010-based genetic tools in combination with faster and higher rates of natural transformation supports the use of an *ago* deletion strain for genetic and engineering studies of *S. elongatus* for research and biotechnology.

## MATERIALS AND METHODS

### Phylogenetic analysis

Multiple alignments of protein sequences and phylogenetic trees were constructed using the software package MEGA version 11 (46). Alignments were generated with MUSCLE (47) implemented in MEGA using default parameters. Positions that can be used reliably in a phylogenetic analysis were extracted with Gblocks 0.91b (48) at settings that allowed the most relaxed selection of blocks. 16S rRNA gene and pAgo phylogenetic trees were inferred by maximum likelihood in MEGA using the Tamura 3-parameter model for the 16S rRNA gene and the Le and Gascuel model for pAgo (49, 50). The trees with the highest log likelihood are shown. The percentage of trees in which the associated taxa clustered together was calculated for each node using bootstrap analyses (*n* = 500) implemented in MEGA. The trees are drawn to scale, with branch lengths measured in the number of substitutions per site.

### Culture conditions

*Escherichia coli* strains were grown at 37°C in lysogeny broth (LB, Lennox) liquid culture or on agar plates, supplemented as needed with 100 µg mL^−1^ ampicillin (Ap), 20 µg mL^−1^ spectinomycin (Sp) plus 20 µg mL^−1^ streptomycin (Sm), 50 µg mL^−1^ spectinomycin (Sp), 15 µg mL^−1^ gentamycin (Gm), 17 µg mL^−1^ chloramphenicol (Cm), 50 µg mL^−1^ kanamycin (Km), 50 µg mL^−1^ nourseothricin (Nt), and 12.5 µg mL^−1^ tetracycline (Tc). Unless noted otherwise, *S. elongatus* PCC 7942 and its derivative strains were grown in BG-11 medium (51) as liquid cultures with continuous orbital shaking (125 rpm) or on BG-11 agar plates (40 mL, 1.5% agar) at 30°C under 12 h:12 h LD cycles or continuous illumination of 200 µmol photons m^−2^ s^−1^ from fluorescent cool white bulbs. Culture media for recombinant cyanobacterial strains were supplemented as needed with 2 µg mL^−1^ Sp plus 2 µg mL^−1^ Sm, 2 µg mL^−1^ Gm, 5 µg mL^−1^ Km, and 5 µg mL^−1^ Nt.

### Molecular methods

PCR amplifications were carried out with Q5 High-Fidelity DNA polymerase (New England BioLabs) according to the manufacturer’s instructions. Plasmid preparations were performed using the QIAprep Spin Miniprep Kit (Qiagen). Restriction digests followed the supplier’s recommendations but with longer incubation times to ensure complete digests. DNA purification/concentration following PCR and restriction digests were performed with DNA Clean & Concentrator TM-5 (Zymo). Nucleic acid concentrations were measured with a NanoDrop 2000c spectrophotometer. Plasmids were constructed by restriction/ligation with NEB Quick Ligase following the manufacturer’s instructions or by Gibson assembly using the GeneArt Seamless Cloning and Assembly Kit (Thermo Fisher) as described previously (28). Plasmids were propagated in *E. coli* DH5α or TOP10 strains with appropriate antibiotics.

### Transformation in *S. elongatus*

Recombinant strains of *S. elongatus* were generally constructed by natural transformation using standard protocols (52). Briefly, liquid cultures were grown to an optical density at 750 nm (OD_750_) of 0.5–0.6. Cells were pelleted by centrifugation at 4500 × *g*, washed once with 10 mM NaCl and once with BG-11 medium, and then resuspended in BG-11 to a concentration of 1 × 10^9^ cells mL^−1^. Samples (200 µL) were incubated in darkness overnight (~16 h) with ~500 ng of plasmid DNA. Transformation reactions were then spread on BG-11 plates with appropriate antibiotics and incubated for 4–5 days under continuous illumination until isolated colonies appeared.

### Conjugation from *E. coli* into *S. elongatus*

Conjugal transfer of plasmid DNA into *S. elongatus* was performed by biparental mating from *E. coli* donor strains following published protocols (53) with minor modifications. Cargo plasmids were electroporated into the conjugal *E. coli* strain AM1359, which harbors conjugal plasmid pRL443, an Ap^R^ Tc^R^ Km^S^ derivative of RP4 (54), and helper plasmid pRL623, which carries a *mob* gene for the conjugal transfer of ColK plasmids and three methylase genes whose products methylate AvaI, AvaII, and AvaIII methylation/restriction sites (53). *E. coli* donor cells were collected by centrifugation at 4500 × *g* for 3 min from 2 mL of 5-mL overnight cultures and resuspended in 1 mL of LB. The cells were washed twice with LB medium to remove residual antibiotics, and after the third centrifugation, the cells were resuspended in 200 µL of LB medium. *S. elongatus* strains (or the RB-Tn-Seq library) were collected by centrifugation at 4500 × *g* for 3 min from 4 mL of a growing culture at an OD_750_ of ~0.5 and resuspended in 1 mL of BG-11 medium. The cells were washed once with BG-11 medium and resuspended in 1 mL of BG-11 medium. The *E. coli* donor and *S. elongatus* strains were combined, pelleted by centrifugation, and finally resuspended in 100-µL BG-11 medium. The cells were then transferred onto a BG-11 agar plate supplemented with 5% (vol/vol) LB and incubated at 30°C under low light intensity (10–20 µmol photons m^−2^ s^−1^) for 24 h. The next day, the cells were washed from the surface of the plate, resuspended in 1 mL of BG-11 medium, collected by centrifugation, resuspended in 200 µL of BG-11 medium, and spread on BG-11 agar plates with the appropriate antibiotics. The plates were incubated under standard growth conditions for 5 to 9 days until isolated colonies appeared.

### Strains and plasmids

Strains, plasmids, and oligonucleotides used in this study are listed in Tables S4 to S6, respectively.

Insertional knockouts of *ago* were generated by transformation of transposon-mutagenized plasmids (8S1-BB11, 8S1-HH1) that carry *S. elongatus* genome fragments flanking a Tn*5* transposon insertion (55). Control strains carrying a Tn*5* transposon at *S. elongatus* chromosomal NS2 or NS3 were generated by natural transformation of the transposon-mutagenized plasmid 8S15-K12 (NS2) (55) or pDE06 (NS3), respectively. To make pDE06, an NS3 plasmid backbone was isolated from pCVD024 by restriction digest with ZraI; a DNA fragment containing a Tn*5* transposon was obtained by PCR from plasmid 8S15-E11 (55) with the primer pair 30_Tn5_GC_F/ 31_Tn5_G5C5_R; then the NS3 plasmid backbone and Tn*5* transposon fragment were assembled by seamless cloning.

Markerless deletions of *ago* were generated by CRISPR-Cas12a editing (35) with pAMT010. To make pAMT010, a DNA fragment coding for the crRNA was obtained by annealing phosphorylated oligonucleotides gRNA_ago_F and gRNA_ago_R and then cloned into pAM5572 digested with AarI by ligation. The resulting plasmid was linearized with KpnI. DNA fragments flanking the *ago* gene were obtained by PCR of *S. elongatus* gDNA using primer pairs Agodel_LA_U805_F/Agodel_LA_U130_R and Agodel_RA_D2084_F/Agodel_RA_D2686_R. Finally, the PCR products were cloned into the linearized plasmid by seamless assembly. Complete segregation of ∆*ago* was verified by PCR, and three independent clones were picked as biological replicates for subsequent experiments.

To complement the *ago* deletion mutation, the coding sequence of *ago* was expressed ectopically at NS2 from a T7 promoter system that depends on a theophylline-inducible T7 RNA polymerase inserted at NS1 (31); however, in the experiments described here, basal expression was used without induction. The *ago* and T7 RNA-polymerase plasmids pAMT007 and pAM5470 were introduced into the *ago* deletion strains AMC2664 and AMC2774 by natural transformation. To make pAMT007, pAM5467 was digested with AflII and SbfI to release the YFP-reporter gene and linearize the plasmid backbone. The *ago* coding sequence was obtained by PCR of *S. elongatus* gDNA using primer pair ago_1534_1F/ago_1534_D2228R and then cloned into the pAM5467 backbone by seamless assembly.

Strains labeled with a fluorescent (yellow or blue) reporter gene were obtained by natural transformation with plasmids pDE03 and pDE17 carrying mVenus and mTagBFP, respectively, each driven by an inducible lacI^q^-P_trc_ promoter system. To make pDE03, the NS1 pAM1303 plasmid backbone carrying a Sp+Sm resistance gene was linearized by restriction digestion with BamHI; a DNA fragment containing *lacI^q^* and *mVenus* driven by the P_trc_ promoter was isolated from pAM4075 by restriction digestion with BglII; and then the fragments were ligated. To make pDE17, we first constructed plasmid pDE04. To make pDE04, the pAM1303 plasmid backbone was linearized by restriction digestion with BamHI, and a DNA fragment containing *lacI^q^* and *mCerulean* driven by the P_trc_ promoter was isolated from pAM4076 by restriction digestion with BglII; the fragments were ligated. *mCerulean* was released from the pDE04 backbone by restriction digestion with BamHI and BsrGI, and a DNA fragment containing mTagBFP was amplified from the iGEM Part: BBa_K592100 by PCR using primers 75_mTagBFP_oRBS_F/76_mTagBFP_oRBS_R and then cloned into the pDE04 backbone by seamless assembly.

To make pAMT025, CYANO-VECTOR devices including a pANS replicon, a pBR322 ori, an Sp+Sm resistance gene, and a YFP-reporter gene were digested from pCVD048, pCVD026, pCVD002, and pCVD044, respectively, and assembled by seamless cloning as described previously (28).

### Quantitative transformation in *S. elongatus*

Cultures of mutant, control, complemented, or WT strains were grown in 100 mL of BG-11 medium bubbled with air at 30°C in LD cycles illuminated with 200 µmol photons m^−1^ s^−1^. The cultures were started at an OD_750_ of ~0.1 and were grown for 3 days until they reached an OD_750_ of ~0.5. At defined time points, 200-µL samples of cells concentrated to 5 × 10^8^  cells mL^−1^ were transformed with 200 ng of suicide plasmids for homologous recombination at a neutral site. At each time point, the cells were incubated with the plasmid DNA in darkness with shaking at 30°C for 3 h; then the reactions were serially diluted, and 25 µL of each dilution was transferred onto agar pads in 12-well plates (3 mL of BG-11 medium solidified with 1.5% agar per well) with the appropriate antibiotics. Alternatively, 4.5 µL of each dilution was spotted on square plates with 50 mL of BG-11 medium solidified with 1.5% agar and the appropriate antibiotics. Plasmid DNA was added 2 h after the onset of darkness; for the experiment presented in Fig. 2A, plasmid DNA was also added 2 h following the return of light. For Fig. S2, plasmid DNA was added at the onset of darkness, and the cells were incubated in darkness for 4 h.

To quantify transformation efficiencies, the 12-well plates were incubated at 30°C under constant illumination to allow the colonies to grow for approximately 5 days. Each plate was photographed, and the pictures were processed with ImageJ and curated manually to produce binary images without background where adjacent colonies were separated. The number of colonies was determined with ImageJ.

### Conjugations from *E. coli* into the *S. elongatus* RB-TnSeq library

Cultures of the RB-TnSeq library (21) were prepared as follows: an aliquot of the library archived at −80°C was thawed for 2 min in a 37°C water bath, then distributed into three flasks containing 100-mL BG-11 with Km, and incubated at 30°C under continuous illumination of 30 µmol photons m^−2^ s^−1^ for 1 day. Then, the cultures were placed on an orbital shaker at 150 rpm under 70 µmol photons m^−2^ s^−1^ of continuous light until they reached an OD_750_ of ∼0.3. A sample of the library was collected prior to conjugation (T0) to determine the population baseline.

Conjugal transfers from an *E. coli* donor strain into the *S. elongatus* RB-TnSeq library were performed as described above. For each experiment, 10 mating assemblages were prepared and spread onto BG-11 agar plates to obtain over 100,000 transconjugant colonies. Three conjugation experiments were preformed to investigate the conjugal transfer of suicide plasmids that recombine into *S. elongatus* chromosomes. For these experiments, a cargo plasmid pAM5329 carrying either a Sp^R^+Sm^R^ gene (*aadA*) that recombines at NS1 (performed twice) or pAM5544 carrying an Nt^R^ gene (*nat7942*) that recombines at NS2 was conjugated into the RB-TnSeq library and then spread onto selective BG-11 agar plates or control plates without antibiotics. Two conjugation experiments were performed to investigate the conjugal transfer and maintenance of broad-host-range plasmids based on RSF1010. For these experiments, a shuttle plasmid containing the RSF1010 replicon (pAM5407) and a shuttle plasmid containing the pANS replicon (pAMT025) were conjugated from an *E. coli* donor strain into the RB-TnSeq library. Both shuttle plasmids carried an Sp^R^+Sm^R^ gene and a YFP reporter gene. The mating assemblages were spread onto selective BG-11 agar plates supplemented with Sp and Sm. The conjugal transfer of pAMT025 served as control.

For each experiment, a minimum of 100,000 colonies were collected, pooled, and stored at −80°C for genomic DNA extraction (52). Then, the barcodes were amplified, sequenced, and quantified in perl v5.18 using previously developed BarSeq protocols (56). The barcodes were curated to keep only barcodes located within the middle 80% of each coding sequence and to keep only the genes represented by at least three barcodes in different positions and with at least 15 T0 reads across replicates. For each gene, a fitness value that represents how the loss of function affected the conjugal transfer and the maintenance of plasmid DNA (through recombination or plasmid replication) and a corresponding statistic were calculated in R version 3.6.0 using methods and scripts developed previously (18, 24, 25). The raw data to perform these analyses are provided at http://golden.ucsd.edu/Rb_TnSeq/.

### Flow cytometry

The abundances of control and *ago* KO cells that were labeled with fluorescent proteins mVenus (yellow cells) and mTagBFP (blue cells) were determined by flow cytometry using a Beckman Coulter CytoFLEX platform (B2-R2-V2). The gains for the FSC and SSC were set to 1,000, with a primary FSC threshold of 100,000 and secondary SSC threshold of 20,000. *S. elongatus* cells were gated based on the autofluorescence of photosynthetic pigments using the APC channel (EX638 and EM660/20) with a gain set to 600. Cells that expressed mVenus were detected using the FITC channel (EX488 and EM525/40) with a gain set to 77, and cells that expressed mTagBFP were detected using the PB450 channel (EX405 and EM450/45) with a gain set to 41. Approximately 20,000 cells per sample were counted. To control for potential growth-altering effects of the different fluorescent reporters, the *ago* mutant and control strains were each marked with each reporter, and competition assays were performed using the reciprocal pairs of strains (e.g., WT-mVenus vs *ago*-mTagBFP and WT-mTagBFP vs *ago*-mVenus). In addition, strains with the same genetic background, but different fluorescent reporters, were competed against each other (e.g., *ago*-mVenus vs *ago*-mTagBFP). As we observed detectable differences in growth depending on the reporter, the numbers of cells recorded at each time point was normalized by the ratio of yellow and blue cells for the corresponding mutation (NS3::Tn*5* or *ago*::Tn*5*) to account for the superior performance of BFP-expressing cells of any genotype.

### Microscopy

Differential interference contrast and fluorescence microscopy were performed on a DeltaVision (Applied Precision, Inc.) microscope system composed of an Olympus IX71 inverted microscope equipped with Olympus UPlanSApo 40×/0.90, 60×/1.35, and 100×/1.40 objectives and a CoolSNAP HQ2/ICX285 camera. Tetramethylrhodamine isothiocyanate filters (EX555/28 and EM617/73) were used to image autofluorescence of photosynthetic pigments. Image acquisitions were performed using Resolve3D softWoRx-Acquire (version 4.0.0). Cell sizes were measured with ImageJ 1.52k.

### Circadian bioluminescence monitoring

Cultures of *S. elongatus* strains expressing the firefly luciferase (*luc*) expressed by the P*_kaiBC_* and P*_purF_* promoters were adjusted to an OD_750_ of 0.2, and 20 µL of cells was distributed in 96-well plates with 270 µL per well of BG11 medium solidified with 1.5% agar and supplemented with 10 µL of 5 mM D-luciferin per well (57). The plates were grown at 30°C for two LD cycles. Then, the plates were placed in a TECAN Infinite 200 Pro equipped with a stacker microplate handling system where plates were maintained at 30°C in constant light (LL). Bioluminescence readings were recorded every 2 h for 6 days. Data were analyzed with BioDare2 using linear detrending (https://biodare2.ed.ac.uk/) (58).

## SUPPLEMENTAL MATERIAL

Data Set S1 mbio.01843-23-s0001.xlsx NS conjugation data.

Data Set S2 mbio.01843-23-s0002.xlsx RSF1010 conjugation data.

Fig. S1 mbio.01843-23-s0003.pdf Alignment of nourseothricin-resistance coding sequences.

Fig. S2 mbio.01843-23-s0004.pdf Natural transformation in WT *S. elongatus* of plasmids pAM5602, pAM5605, and pAM5607.

Fig. S3 mbio.01843-23-s0005.pdf Secondary structure of SeAgo predicted using AlphaFold.

Supplemental tables mbio.01843-23-s0006.pdf Tables S1-S3.

Supplemental tables mbio.01843-23-s0007.pdf Tables S4-S6.

ASM does not own the copyrights to Supplemental Material that may be linked to, or accessed through, an article. The authors have granted ASM a non-exclusive, world-wide license to publish the Supplemental Material files. Please contact the corresponding author directly for reuse.
