# Supplementary material for: Synechococcus elongatus Argonaute reduces natural transformation efficiency and provides immunity against exogenous plasmids
Source: mBio. 2023 Oct 4;14(5):e01843-23. doi: 10.1128/mbio.01843-23 (PMC10653904; doi:10.1128/mbio.01843-23)
Supplement: Fig. S1 — Alignment of nourseothricin-resistance coding sequences. [file mbio.01843-23-s0003.pdf]

nat7120
nat7942

```
             *         *         *         *         *         *         *         *         *         *
  1 ATGACTACTTTAGATGATACTGCTTATCGCTATCGCACAAGTGTTCCAGGTGATGCTGAAGCTATTGAAGCATTAGATGGTAGTTTTACTACTGATACTG
    |||||#||##|#||||||||#||#||#|||||#|||||#||#|||||#||||||||#|||||#||#|||||##|#|||||#||#|||||#||#|||||#|
  1 ATGACCACCCTGGATGATACCGCCTACCGCTACCGCACCAGCGTTCCCGGTGATGCCGAAGCCATCGAAGCCCTGGATGGCAGCTTTACCACCGATACCG
             *         *         *         *         *         *         *         *         *         *

             *         *         *         *         *         *         *         *         *         *
101 TGTTTCGCGTTACTGCTACTGGTGATGGTTTTACTTTACGTGAAGTTCCAGTTGATCCACCCTTAACTAAAGTTTTTCCCGATGATGAAAGTGATGATGA
    ||||||||||#||#||#||#||||||||#|||||##|#||#|||||#||#||#|||||#||##|#||#||||#||||||||||||||||||||||||||
101 TGTTTCGCGTGACCGCCACGGGTGATGGCTTTACCCTGCGCGAAGTGCCCGTCGATCCCCCTCTGACCAAAGTGTTTCCCGATGATGAAAGTGATGATGA
             *         *         *         *         *         *         *         *         *         *

             *         *         *         *         *         *         *         *         *         *
201 ATCTGATGCTGGTGAAGATGGTGATCCAGATAGTCGTACTTTTGTTGCTTATGGTGATGATGGTGATTTAGCTGGTTTTGTGGTTGTGAGTTATAGTGGT
    |||#||||||||#||||||||#|||||#|||||#||#||#|||||#||#||#|||||||||||#|||#|#|||||#||||||||#|||||#||#||#||#
201 ATCGGATGCTGGCGAAGATGGCGATCCCGATAGCCGCACCTTTGTGGCCTACGGTGATGATGGCGATCTGGCTGGCTTTGTGGTGGTGAGCTACAGCGGC
             *         *         *         *         *         *         *         *         *         *

             *         *         *         *         *         *         *         *         *         *
301 TGGAATCGTCGTTTAACCGTTGAAGATATTGAAGTTGCTCCAGAACATCGTGGTCATGGTGTTGGTCGTGCTTTAATGGGTTTAGCTACTGAATTTGCTC
    ||||||||#||#||#|||||#||||||||||||||#|||||#|||||#||#|||||#||#||||||||#|||#|#|||||##|#||#||#||||||||||
301 TGGAATCGCCGCTTGACCGTGGAAGATATTGAAGTGGCTCCCGAACACCGCGGTCACGGCGTTGGTCGCGCTCTGATGGGCCTGGCCACCGAATTTGCTC
             *         *         *         *         *         *         *         *         *         *

             *         *         *         *         *         *         *         *         *         *
401 GTGAACGTGGTGCTGGTCATTTATGGTTAGAAGTGACTAATGTTAACGCTCCCGCTATTCATGCTTATCGTCGTATGGGTTTTACCTTGTGTGGTTTAGA
    |#|||||#||||||||#||##|#|||#|#||||||||#||#|#|#|||||||||||||#||#||#|||||#||#|||||#||#|||#|||||||##|#||
401 GCGAACGCGGTGCTGGCCACCTGTGGCTGGAAGTGACCAACGTGAACGCTCCCGCTATCCACGCCTATCGCCGCATGGGCTTCACCCTGTGTGGCCTGGA
             *         *         *         *         *         *         *         *         *         *

             *         *         *         *         *         *         *
501 TACCGCCTTATATGATGGTACTGCTAGTGATGGTGAACAAGCCTTATATATGAGTATGCCATGTCCATAA 570
    ||||||##|#||#|||||#||#||#|||||||#|||||#|||#|#||#||||#|||||#||#||#|||
501 TACCGCTCTGTACGATGGCACCGCCAGTGATGGCGAACAGGCCCTGTACATGAGCATGCCCTGCCCCTAA 570
             *         *         *         *         *         *         *
```

```
Matches(|):455
Mismatches(#):115
Gaps( ):0
Unattempted(.):0
```

FIG S1 Alignment of the nourseothricin-resistance coding sequences codon-optimized for *Anabeana* PCC 7120 and *S. elongatus* PCC 7942.
