# Supplementary material for: Synechococcus elongatus Argonaute reduces natural transformation efficiency and provides immunity against exogenous plasmids
Source: mBio. 2023 Oct 4;14(5):e01843-23. doi: 10.1128/mbio.01843-23 (PMC10653904; doi:10.1128/mbio.01843-23)
Supplement: Fig. S2 — Natural transformation in WT S. elongatus of plasmids pAM5602, pAM5605, and pAM5607. [file mbio.01843-23-s0004.pdf]

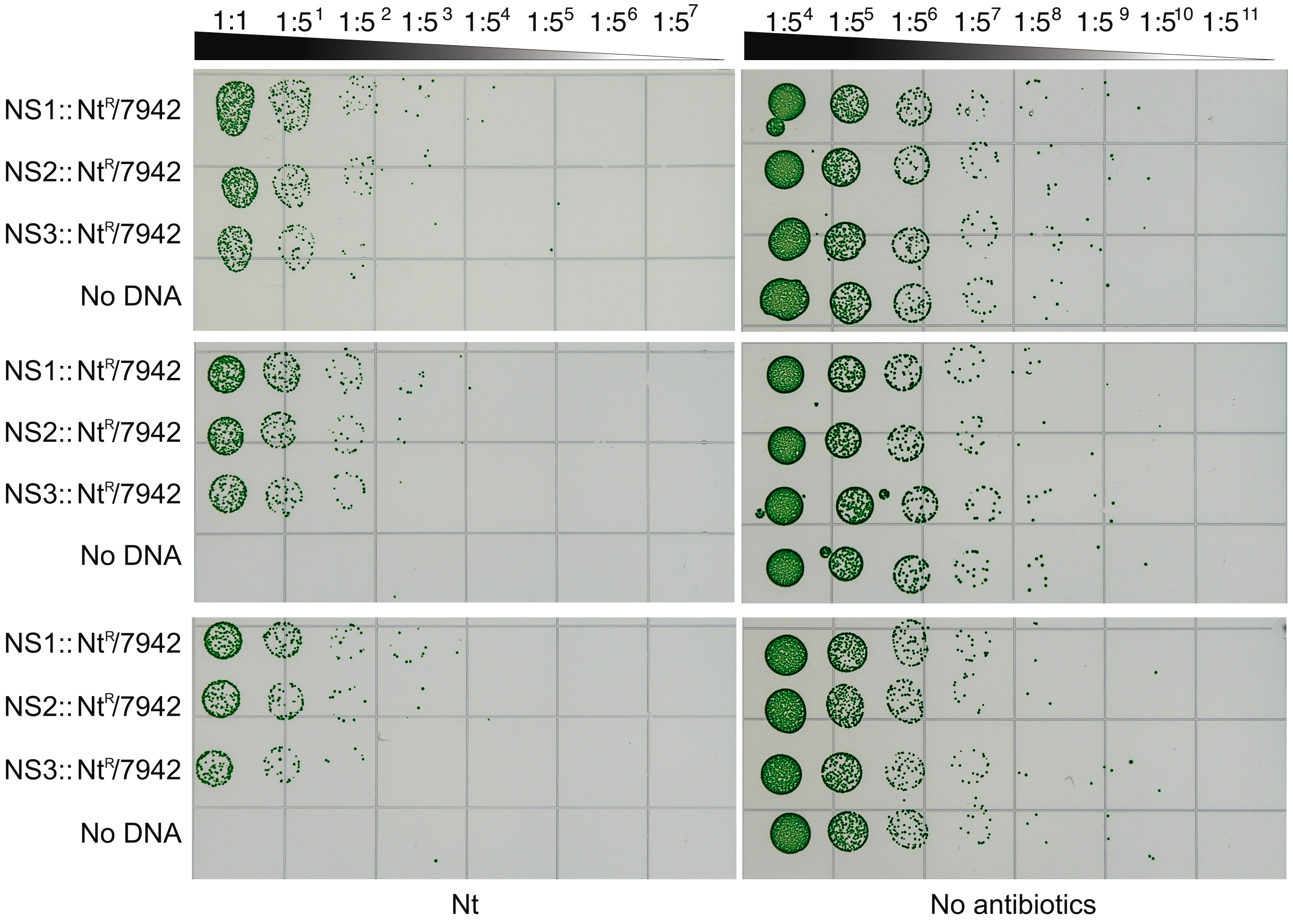

FIG S2 Natural transformation in WT *S. elongatus* of plasmids pAM5602, pAM5605, and pAM5607 that carry the same Nt^R/7942 gene but recombine at 3 different neutral sites: NS1::Nt^R/7942, NS2::Nt^R/7942, and NS3::Nt^R/7942, respectively. Nt, nourseothricin.
