# Supplementary material for: Synechococcus elongatus Argonaute reduces natural transformation efficiency and provides immunity against exogenous plasmids
Source: mBio. 2023 Oct 4;14(5):e01843-23. doi: 10.1128/mbio.01843-23 (PMC10653904; doi:10.1128/mbio.01843-23)
Supplement: Fig. S3 — Secondary structure of SeAgo predicted using AlphaFold. [file mbio.01843-23-s0005.pdf]

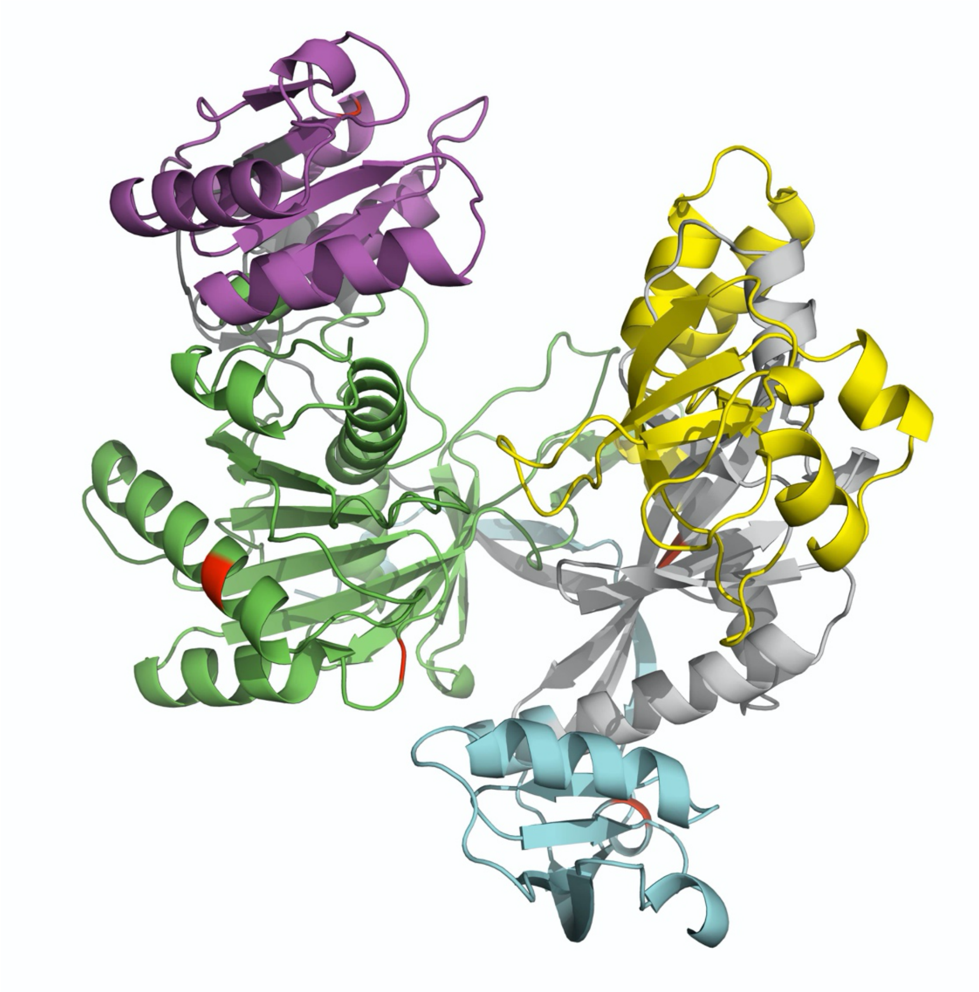

FIG S3 Secondary structure of SeAgo predicted using AlphaFold (1, 2). The location of second-site mutations that enabled the maintenance of RSF1010-based plasmids (Fig. 4) are shown in red. The SeAgo domains were colored as followed. N: cyan, L1: light gray, PAZ: yellow, L2: dark gray, MID: magenta, and PIWI: green.

References

1. Jumper J, Evans R, Pritzel A, Green T, Figurnov M, Ronneberger O, Tunyasuvunakool K, Bates R, Zidek A, Potapenko A, Bridgland A, Meyer C, Kohl SAA, Ballard AJ, Cowie A, Romera-Paredes B, Nikolov S, Jain R, Adler J, Back T, Petersen S, Reiman D, Clancy E, Zielinski M, Steinegger M, Pacholska M, Berghammer T, Bodenstein S, Silver D, Vinyals O, Senior AW, Kavukcuoglu K, Kohli P, Hassabis D. 2021. Highly accurate protein structure prediction with AlphaFold. Nature 596:583-589.

2. Varadi M, Anyango S, Deshpande M, Nair S, Natassia C, Yordanova G, Yuan D, Stroe O, Wood G, Laydon A, Zidek A, Green T, Tunyasuvunakool K, Petersen S, Jumper J, Clancy E, Green R, Vora A, Lutfi M, Figurnov M, Cowie A, Hobbs N, Kohli P, Kleywegt G, Birney E, Hassabis D, Velankar S. 2022. AlphaFold Protein Structure Database: massively expanding the structural coverage of protein-sequence space with high-accuracy models. Nucleic Acids Res 50:D439-D444.
