# Supplementary material for: Synechococcus elongatus Argonaute reduces natural transformation efficiency and provides immunity against exogenous plasmids
Source: mBio. 2023 Oct 4;14(5):e01843-23. doi: 10.1128/mbio.01843-23 (PMC10653904; doi:10.1128/mbio.01843-23)
Supplement: Supplemental tables — Tables S1-S3. [file mbio.01843-23-s0006.pdf]

Table S1. Sequencing results listing polymorphisms for *S. elongatus* AMC2751 as predicted using breseq (1).

position	polymo.	annotation	gene	description
92,978	T→C	Q121R (CAG→CGG)	Synpcc7942_0095 ←	response regulator transcription factor
924,962	T→C	L295P (CTG→CCG)	Synpcc7942_0918 →	long-chain-fatty-acid CoA ligase
925,952	C→T	T625I (ACC→ATC)	Synpcc7942_0918 →	long-chain-fatty-acid CoA ligase
1,527,174	Δ1 bp	intergenic (-220/-543)	Synpcc7942_1475 ← / → Synpcc7942_1476	sodium-dependent bicarbonate transport family permease/hypothetical protein
1,593,099	Δ2,190 bp		[Synpcc7942_1534]	Piwi domain-containing protein pAgo

Table S2. Sequencing results listing polymorphisms for *S. elongatus* AMC2752 as predicted using breseq (1).

position	polymo.	annotation	gene	description
92,978	T→C	Q121R (CAG→CGG)	Synpcc7942_0095 ←	response regulator transcription factor
924,962	T→C	L295P (CTG→CCG)	Synpcc7942_0918 →	long-chain-fatty-acid CoA ligase
925,952	C→T	T625I (ACC→ATC)	Synpcc7942_0918 →	long-chain-fatty-acid CoA ligase
1,527,174	Δ1 bp	intergenic (-220/-543)	Synpcc7942_1475 ← / → Synpcc7942_1476	sodium-dependent bicarbonate transport family permease/hypothetical protein
1,593,099	Δ2,190 bp		[Synpcc7942_1534]	Piwi domain-containing protein pAgo

Table S3. Sequencing results listing polymorphisms for *S. elongatus* AMC2665 as predicted*[1] using breseq (1).

position	polymo.	freq	annotation	gene	description
92,978	T→C	90.6%	Q121R (CAG→CGG)	*Synpcc7942_0095* ←	response regulator transcription factor
850,202	C→G	11.9%	intergenic (+97/-31)	*Synpcc7942_0853*→ / → *Synpcc7942_0854*	LL-diaminopimelate aminotransferase/hypothetical protein
850,383	T→G	9.8%	S51A (TCG→GCG)	*Synpcc7942_0854* →	hypothetical protein
924,962	T→C	100%	L295P (CTG→CCG)	*Synpcc7942_0918* →	long-chain-fatty-acid CoA ligase
924,992	T→A	38.6%	V305E (GTG→GAG)	*Synpcc7942_0918* →	
925,952	C→T	37.1%	T625I (ACC→ATC)	*Synpcc7942_0918* →	
1,527,150	A→G	10.2%	intergenic (-196/-567)	*Synpcc7942_1475* ← / → *Synpcc7942_1476*	sodium-dependent bicarbonate transport family permease / hypothetical protein
1,527,174	Δ1 bp	*[2]	intergenic (-220/-543)	Synpcc7942_1475 ← / → Synpcc7942_1476	
1,527,190	C→A	32.3%	intergenic (-236/-527)	*Synpcc7942_1475* ← / → *Synpcc7942_1476*	
1,572,350	C→A	23.3%	intergenic (-70/-136)	*Synpcc7942_1519* ← / → *Synpcc7942_1520*	histidinol dehydrogenase / 30S ribosomal protein S20
2,195,091:1	+A	48.6%	coding (265/1164 nt)	*Synpcc7942_2114* →	two component system sensor histidine kinase SasA

*[1] Predictions were performed using the mixed population mode available in breseq.
*[2] Not predicted as a polymorphism using breseq, but this variant was found in the raw sequencing data.

References

1. Deatherage DE, Barrick JE. 2014. Identification of mutations in laboratory-evolved microbes from next-generation sequencing data using breseq. Methods Mol Biol 1151:165-88.
