# Supplementary material for: Synechococcus elongatus Argonaute reduces natural transformation efficiency and provides immunity against exogenous plasmids
Source: mBio. 2023 Oct 4;14(5):e01843-23. doi: 10.1128/mbio.01843-23 (PMC10653904; doi:10.1128/mbio.01843-23)
Supplement: Supplemental tables — Tables S4-S6. [file mbio.01843-23-s0007.pdf]

Table S4. Strains used in this study

Strain	Description	Resistance	Source
Escherichia coli			
DH10B	Cloning strain	Sm	Laboratory collection
DH5α	Cloning strain		Laboratory collection
AM1359	Conjugal strain, *E. coli* DH10B carrying helper plasmid pRL623 and conjugal plasmid pRL443 helper.	Ap, Tc, Cm, Sm	(1)
**Cyanobacteria**			
LB1.0/2.0	*S. elongatus* RB-TnSeq library	Km	(2)
AMC06	Wild-type strain of *S. elongatus* PCC 7942 archived in 1988 and refrozen several times.		Laboratory collection
AMC2302	*S. elongatus* PCC 7942 cured of its small plasmid pANS		(3)
AMC2691	NS1::P_{trc}-*mVenus* / NS3::Tn*5* (pDE06)	Sp, Sm, Km	This study
AMC2695	NS1::P_{trc}-*mVenus* (pDE03) / *ago*::Tn*5* (8S1-HH1)	Sp, Sm, Km	This study
AMC2699	NS1::P_{trc}-*mTagBFP* (pDE17) / NS3::Tn*5* (pDE06)	Sp, Sm, Km	This study
AMC2703	NS1::P_{trc}-*mTagBFP* (pDE17) / *ago*::Tn*5* (8S1-HH1)	Sp, Sm, Km	This study
AMC2664	AMC06, Δ*ago*		This study
AMC2774	AMC2302, Δ*ago*		This study
AMC2775	AMC2774, (NS2::P_{T7}-*ago* + NS1::$_{PconII*}$-RSWB-*T7RNAP*)	Gm, Sp, Sm	This study
AMC2776	AMC2664, (NS2::P_{T7}-*ago* + NS1::$_{PconII*}$-RSWB-*T7RNAP*)	Gm, Sp, Sm	This study
AMC2633	S7942_NS2::(cat-P_{kaiBC}-*luc*), *sasA* – N93E	Cm	(4)
AMC2610	S7942_NS2::(cat-P_{kaiBC}-*luc*), *rpaA* – R4L	Cm	Laboratory collection
AMC2636	S7942_NS2::(cat-P_{kaiBC}-*luc*), *sasA* – H28A	Cm	(4)
AMC2635	S7942_NS2::(cat-P_{kaiBC}-*luc*), *sasA* – Q101A	Cm	(4)
AMC2634	S7942_NS2::(cat-P_{kaiBC}-*luc*), *sasA* – Q97A	Cm	(4)
AMC2632	S7942_NS2::(cat-P_{kaiBC}-*luc*), *sasA* – N93A	Cm	(4)
AMC2631	S7942_NS2::(cat-P_{kaiBC}-*luc*), *sasA* – Q94A	Cm	(4)
AMC2649	S7942_NS2::(cat-P_{kaiBC}-*luc*), *sasA* – Q97E	Cm	(4)
AMC2665	Wild-type strain of *S. elongatus* PCC 7942 frozen in 1988 and refrozen in 2021.		This study
AMC2751	AMC2665, Δ*ago*		This study
AMC2752	AMC2665, Δ*ago*		This study
AMC2747	AMC2665, S7942_NS2::(*cat*-P_{kaiBC}-*luc*)	Cm	This study
AMC2748	AMC2665, S7942_NS1::(*aadA*-P_{purF}-*luc*)	Sp, Sm	This study
AMC2749	AMC2752, S7942_NS2::(*cat*-P_{kaiBC}-*luc*)	Cm	This study
AMC2750	AMC2752, S7942_NS1::(*aadA*-P_{purF}-*luc*)	Sp, Sm	This study

Table S5. Plasmids used in this study

Name	Description / Genotype	Antibiotic resistance	Source
**PLASMIDS – parts and backbones**			
pAM5467	S7942_NS2::(*aacC1*-P_{T7}-*YFP*)	Tc, Gm	(5)
pAM5572	Broad host range plasmid for genome editing using CRISPR-Cas12a.	Sp, Sm	(6)
pCVD002	Sp^R+Sm^R gene cassette (CYANO-VECTOR donor plasmid)	Ap, Sp, Sm	(7)
pCVD024	S7942_NS3 (CYANO-VECTOR donor plasmid)	Ap	(7)
pCVD026	pBR322 ori (CYANO-VECTOR donor plasmid)	Ap	(7)
pCVD044	P_{conII}-*YFP* (CYANO-VECTOR donor plasmid)	Ap	(7)
pCVD048	*S. elongatus* pANS replicon (CYANO-VECTOR donor plasmid)	Ap	(7)
pAM1303	S7942_NS1::*aadA*	Sp, Sm	(8)
pAM4075	Plasmid carrying *lacIq*-P_{trc}-*mVenus*	Ap	Laboratory collection
pAM4076	Plasmid carrying *lacIq*-P_{trc}-*mCerulean*	Ap	Laboratory collection
BBa_K592100	iGEM part containing *mTagBFP*		(9)
**PLASMIDS – knockouts, controls, and complemented strains**			
8S1-BB11	*S. elongatus* gDNA library clone with a Tn*5* insertion in *ago*	Km	(10)
8S1-HH1	*S. elongatus* gDNA library clone with a Tn*5* insertion in *ago*	Km	(10)
8S15-E11	*S. elongatus* gDNA library clone with a Tn*5* insertion at NS3	Km	(10)
8S15-K12	*S. elongatus* gDNA library clone with a Tn*5* insertion at NS2	Km	(10)
pAMT010	pAM5572 with SeAgo guide and *ago* flanking sequences for homologous recombination	Sp, Sm	This study
pDE06	S7942_NS3::Tn*5*	Km	This study
pAM5470	S7942_NS1::(*aadA*-P_{conII*}- RSWB-*T7RNAP*)	Sp, Sm	(5)
pAMT007	S7942_NS2::(*aacC1*-P_{T7}-*ago*)	Gm	This study
**PLASMIDS – assays**			
pAM2105	S7942_NS2::(*cat*-P_{kaiBC}-*luc*)	Cm	Laboratory collection
pAM2225	S7942_NS1::(*aadA*-P_{purF}-*luc*)	Sp, Sm	Laboratory collection
pAM4920	S7942_NS1-Tc::(*nat7120*-P_{conII}-*YFP*)	Nt, Tc	(7)
pAM4921	S7942_NS1-Tc::(*nat7942*-P_{conII}-*YFP*)	Nt, Tc	(7)
pAM4970	RSF1010::(*nat7942*-P_{psbAI}-*yemGFP*)	Nt	(7)
pAM4971	S7942_pANS::(*nat7942*-P_{psbAI}-*yemGFP*)	Nt	(7)
pAM5328	S7942_NS3::*aacC1*	Gm	(11)
pAM5329	S7942_NS1::*aadA*	Sp, Sm	(11)
pAM5407	RSF1010-*aadA*-P_{conII*}-*YFP*	Sp, Sm	(7)
pAM5544	S7942_NS2::*nat7942*	Nt	(12)
pAM5554	S7942_NS3::*nat7942*	Nt	(12)
pAM5602	S7942_NS1::(*nat7942*-P_{conII}-*yemGFP*)	Nt, Tc	This study
pAM5605	S7942_NS2::(*nat7942*-P_{conII}-*yemGFP*)	Nt, Tc	This study
pAM5607	S7942_NS3::(*nat7942*-P_{conII}-*yemGFP*)	Nt, Tc	This study
pAMT025	pANS-*aadA*-P_{conII}-*YFP*	Sp, Sm	This study
pDE04	S7942_NS1::(*aadA*_P_{trc}-*mCerulean*)	Sp, Sm	This study
pDE03	S7942_NS1::(*aadA*_P_{trc}-*mVenus*)	Sp, Sm	This study
pDE17	S7942_NS1::(*aadA*_P_{trc}-*mTagBFP*)	Sp, Sm	This study

Table S6. Oligonucleotides used in this study

Name	Sequence (5' to 3')
gRNA_ago_F	AGATATGCTTTCACATGGATCTAC
gRNA_ago_R	AGACGTAGATCCATGTGAAAGCAT
Agodel_LA_U805_F	TTTAATGCGGTAGTTGGTACGTTCAAAACCTATGCGTTGCAGC
Agodel_LA_U130_R	CAAGAGCGGAAACCACTCGCTGCAGTCTTAACGTTGCTGCCAGAGG
Agodel_RA_D2084_F	CCTCTGGCAGCAACGTTAAGACTGCAGCGAGTGGTTTCCGCTCTTG
Agodel_RA_D2686_R	GCCCCCGATGTCGACGGTACCAGGAGTGGCTGAGTCACTGGG
ago_1534_1F	GGGAGAATTTAAATAAAGGAGGTCTTAAGATGGATCTACTATCAAATTTGCGGCG
ago_1534_D2228R	CAGGATGGCCTTCTCCTGCACGAGTACTGAGCCATCACGCTTAGAC
30_Tn5_GC_F	ATCGCGCGCGCGCGCGCGGACCTGTCTCTTATACACATCTC
31_Tn5_G5C5_R	ATCCCCCCGGGGGCCCCCGATCTGTCTCTTATACACATCTC
75_mTagBFP_oRBS_F	GGAATTCGAGCTCGGTACCCGGGAGAAGGAGGTTTCTAGATGAGCGAACTGATCAAAGAG
76_mTagBFP_oRBS_R	GCCAAGCTTGCATGCCTGCAGGTCGACTTATTAATTCAGTTTATGACCCAG

References

1. Elhai J, Vepritskiy A, Muro-Pastor AM, Flores E, Wolk CP. 1997. Reduction of conjugal transfer efficiency by three restriction activities of *Anabaena* sp. strain PCC 7120. J Bacteriol 179:1998-2005.

2. Rubin BE, Wetmore KM, Price MN, Diamond S, Shultzaberger RK, Lowe LC, Curtin G, Arkin AP, Deutschbauer A, Golden SS. 2015. The essential gene set of a photosynthetic organism. Proc Natl Acad Sci U S A 112:E6634-43.

3. Chen Y, Taton A, Go M, London RE, Pieper LM, Golden SS, Golden JW. 2016. Self-replicating shuttle vectors based on pANS, a small endogenous plasmid of the unicellular cyanobacterium *Synechococcus elongatus* PCC 7942. Microbiology (Reading) 162:2029-2041.

4. Chavan AG, Swan JA, Heisler J, Sancar C, Ernst DC, Fang M, Palacios JG, Spangler RK, Bagshaw CR, Tripathi S, Crosby P, Golden SS, Partch CL, LiWang A. 2021. Reconstitution of an intact clock reveals mechanisms of circadian timekeeping. Science 374:eabd4453.

5. Roulet J, Taton A, Golden JW, Arabolaza A, Burkart MD, Gramajo H. 2018. Development of a cyanobacterial heterologous polyketide production platform. Metab Eng 49:94-104.

6. Taton A, Ecker A, Diaz B, Moss NA, Anderson B, Reher R, Leao TF, Simkovsky R, Dorrestein PC, Gerwick L, Gerwick WH, Golden JW. 2020. Heterologous Expression of Cryptomaldamide in a Cyanobacterial Host. ACS Synth Biol 9:3364-3376.

7. Taton A, Unglaub F, Wright NE, Zeng WY, Paz-Yepes J, Brahamsha B, Palenik B, Peterson TC, Haerizadeh F, Golden SS, Golden JW. 2014. Broad-host-range vector system for synthetic biology and biotechnology in cyanobacteria. Nucleic Acids Res 42:e136.

8. Andersson CR, Tsinoremas NF, Shelton J, Lebedeva NV, Yarrow J, Min H, Golden SS. 2000. Application of bioluminescence to the study of circadian rhythms in cyanobacteria. Methods Enzymol 305:527-42.

9. Subach OM, Gundorov IS, Yoshimura M, Subach FV, Zhang J, Gruenwald D, Souslova EA, Chudakov DM, Verkhusha VV. 2008. Conversion of red fluorescent protein into a bright blue probe. Chem Biol 15:1116-24.

10. Chen Y, Holtman CK, Taton A, Golden SS. 2012. Functional Analysis of the *Synechococcus elongatus* PCC 7942 Genome, p 119-137, Functional Genomics and Evolution of Photosynthetic Systems, vol 33. Springer Netherlands, Dordrecht.

11. Taton A, Ma AT, Ota M, Golden SS, Golden JW. 2017. NOT Gate Genetic Circuits to Control Gene Expression in Cyanobacteria. ACS Synth Biol 6:2175-2182.

12. Taton A, Erikson C, Yang Y, Rubin BE, Rifkin SA, Golden JW, Golden SS. 2020. The circadian clock and darkness control natural competence in cyanobacteria. Nat Commun 11:1688.
